# Associations of the *PON1* rs854560 polymorphism with plasma lipid levels: a meta-analysis

**DOI:** 10.1186/s12944-018-0924-0

**Published:** 2018-12-03

**Authors:** Zhi Luo, Shujin Li, Irfan Muhammad, Md Rezaul Karim, Yongyan Song

**Affiliations:** 1 Department of Cardiology, Affiliated Hospital of North Sichuan Medical College, Nanchong, 637000 People’s Republic of China; 2 School of Continuing Education, North Sichuan Medical College, Nanchong, 637000 People’s Republic of China; 3 Department of Neurology, Taihe Hospital of Hubei University of Medicine, Shiyan, Hubei 442000 People’s Republic of China; 4 Department of Medical Biochemistry, and Nanchong Key Laboratory of Metabolic Drugs and Biological Products, North Sichuan Medical College, Nanchong, 637000 People’s Republic of China

**Keywords:** Paraoxonase 1, Polymorphism, Lipids, Meta-analysis

## Abstract

**Background:**

Previous studies have investigated the associations of paraoxonase 1 (*PON1*) rs854560 polymorphism with plasma lipid levels, but the results are inconclusive. This meta-analysis aimed to clarify the associations of the rs854560 polymorphism with plasma lipid levels.

**Methods:**

A comprehensive search of the literature was carried out by using the databases which include Medline, Google Scholar, Web of Science, Embase, Cochrane Library, China National Knowledge Infrastructure (CNKI), Wanfang and VIP database up till August 2018. The pooled standardized mean difference (SMD) with 95% confidence interval (CI) was used to assess the differences in lipid levels between the genotypes. Begg’s funnel plots and Egger’s test were used to examine the publication bias.

**Results:**

A total of 41 studies (22,844 subjects) were identified for the associations of rs854560 polymorphism with plasma lipid levels. The M carriers had lower levels of high-density lipoprotein Cholesterol (HDL-C) (SMD = − 0.15, 95% CI = − 0.23--0.07, *P* < 0.01) and apolipoprotein A-I (APOA1) (SMD = − 0.67, 95% CI = − 0.93--0.41, P < 0.01) than the non-carriers. Subgroup analysis by ethnicity revealed that the effect on HDL level was significant in Caucasians and the subjects of other ethnic origins. No publication bias was detected in this meta-analysis.

**Conclusions:**

The meta-analysis suggests that the *PON1* rs854560 polymorphism is associated with a lower HDL-C level in Caucasians and subjects of other ethnic origins.

**Electronic supplementary material:**

The online version of this article (10.1186/s12944-018-0924-0) contains supplementary material, which is available to authorized users.

## Introduction

Coronary heart disease (CHD) is the leading cause of death in China and most of the developed countries [1]. While the interactions between genetic and environmental factors determine the pathogenesis of this disease, the report shows that dyslipidemia ranks as one of the most important risk factors accounting for at least 50% of the population attributable risk [2]. Dyslipidemia is characterized by elevated levels of triglycerides (TG), total cholesterol (TC) and low-density lipoprotein cholesterol (LDL-C) as well as reduced levels of high-density lipoprotein cholesterol (HDL-C) in circulation. Over the years, several investigations have been carried out on the genetic polymorphism/mutation that affects plasma lipid levels. The results, however, have been inconclusive primarily due to small sample size, ethnicity and difference in health conditions.

High-density lipoprotein (HDL) has been shown to play a critical role in reverse cholesterol transport (RCT) by eliciting cholesterol efflux from macrophage foam cells which prevents the progression of atherosclerotic lesions and induces the regression of existing plaques [3]. HDL-C has also been primarily associated with a protein, apolipoprotein A-I (APOA1) and the over-expression of APOA1 has equally been found to reduce atherosclerosis in mice. Along the same line, Paraoxonase 1 (PON1), an enzyme that hydrolyses aryl esters, phosphate esters and lactones has been shown to be associated with apoA-1 and HDL-C. According to Akbas et al. [4] HDL-associated PON1 is considered to be a major anti-atherosclerosis component of HDL, as it inhibits the oxidation of low-density lipoprotein (LDL) and promotes cholesterol efflux from macrophage foam cells [4–6].

The *PON1* gene is located on the long arm of human chromosome 7 (7q21–22), containing 9 exons and 8 introns. Although some studies have demonstrated that *PON1* gene rs854560 polymorphism was significantly associated with CHD [7–9], whether this polymorphism is associated with dyslipidemia remains to be examined. A number of researches have investigated the associations of this polymorphism with plasma lipid levels, but the results were inconsistent and inconclusive. In some of these studies, the M allele of the rs854560 polymorphism was reported to be significantly associated with higher levels of TG [10–12], TC [13, 14], LDL-C [13–15], and lower levels of HDL-C [14, 16] and apoA-I [16] while reports from other studies differ [17–21]. Hence, we conducted this meta-analysis to clarify the associations of the *PON1* rs854560 polymorphism and the different plasma lipid levels by using a larger sample size and to put into consideration ethnicity and disease condition particularly atherosclerosis.

## Methods

### Literature search

A comprehensive search of the literature was carried out by using databases which include Medline, Google Scholar, Web of Science, Embase, Cochrane Library, China National Knowledge Infrastructure (CNKI), Wanfang and VIP database (up till August 2018). The terms “paraoxonase 1” or “PON1”; “polymorphism” or “mutation” or “variant” or “SNP” or “rs854560” or “L55 M”; “blood lipid” or “serum lipid” or “lipids” were used for the search. The variables were limited to TG, TC, LDL-C, HDL-C and APOA1. The languages of the articles were limited to English and Chinese. All references cited in the included articles were reviewed to check for published works that were not indexed by Medline, Google Scholar, Web of Science, Embase, Cochrane Library, CNKI, Wanfang and VIP database.

### Inclusion criteria

Studies that fulfill the following criteria were included: (1) studies in which the mean serum lipid values and standard deviations (SD) or standard errors (SE) by the rs854560 genotypes were available; (2) data reported on at least one of the five variables (TG, TC, LDL-C, HDL-C and APOA1); (3) data reported on fasting lipid variables; (4) pre-intervention baseline data that were used for interventional studies. Reports with incomplete data, studies based on pedigree data, case reports, review articles, abstracts and animal studies were excluded from the meta-analysis.

### Data extraction

The studies that do not meet the inclusion criteria were excluded after being reviewed independently by two reviewers. All data were double-checked, compared after extraction and disagreements between reviewers were discussed and resolved. From each paper, the following information was collected: first author’s name, year of publication, average age, country, ethnicity, gender, health condition and the mean of serum lipid levels and SD by genotypes. If a paper’s data were unconvincing, we tried to contact the correspondent author by e-mail. All the information was extracted using a standardized data collection form.

### Data analysis

All statistical tests were two-sided and conducted by the STATA software package (Version 10, Stata Corporation, College Station, TX). *P*-value smaller than 0.05 for any test or model was considered to be statistically significant. Standardized mean difference (SMD) with 95% confidence interval (CI) was used for the meta-analysis. A fixed effect model (a Mantel-Haenszel method) was used to evaluate the results when heterogeneity among studies investigated by Cochrane Q statistic was not significant (I^2^ ≤ 50%, *P* > 0.05). Otherwise, the random effect model (DerSimonian and Laird) was used [22]. Where there was significant heterogeneity among studies, we performed the Galbraith plot to detect potential sources of heterogeneity. Since the results of most of the included studies were reported in a dominant way (LM + MM vs LL), a dominant model was employed to ensure adequate statistical power. Subgroup analyses were performed by ethnicity, gender and health condition. The ethnic subgroup was defined as Caucasian, Asian, and subjects of other ethnic origins. Health condition subgroup was defined as CHD patients, type 2 diabetes mellitus (T2DM) patients and healthy/control subjects. When data were presented for more than one subpopulation (e.g., female or male subjects, the subjects with CHD or T2DM, the subjects from different ethnicities) in one article, each subpopulation was treated as a separate comparison. Hardy-Weinberg equilibrium (HWE) was assessed by Fisher’s exact test and a *P*-value < 0.05 was considered statistically significant. Possible publication bias was tested by Begg’s funnel plots and Egger’s test using *P* < 0.05 to indicate the presence of potential publication bias.

## Result

### Selection and characteristics of studies

**Fig. 1:**
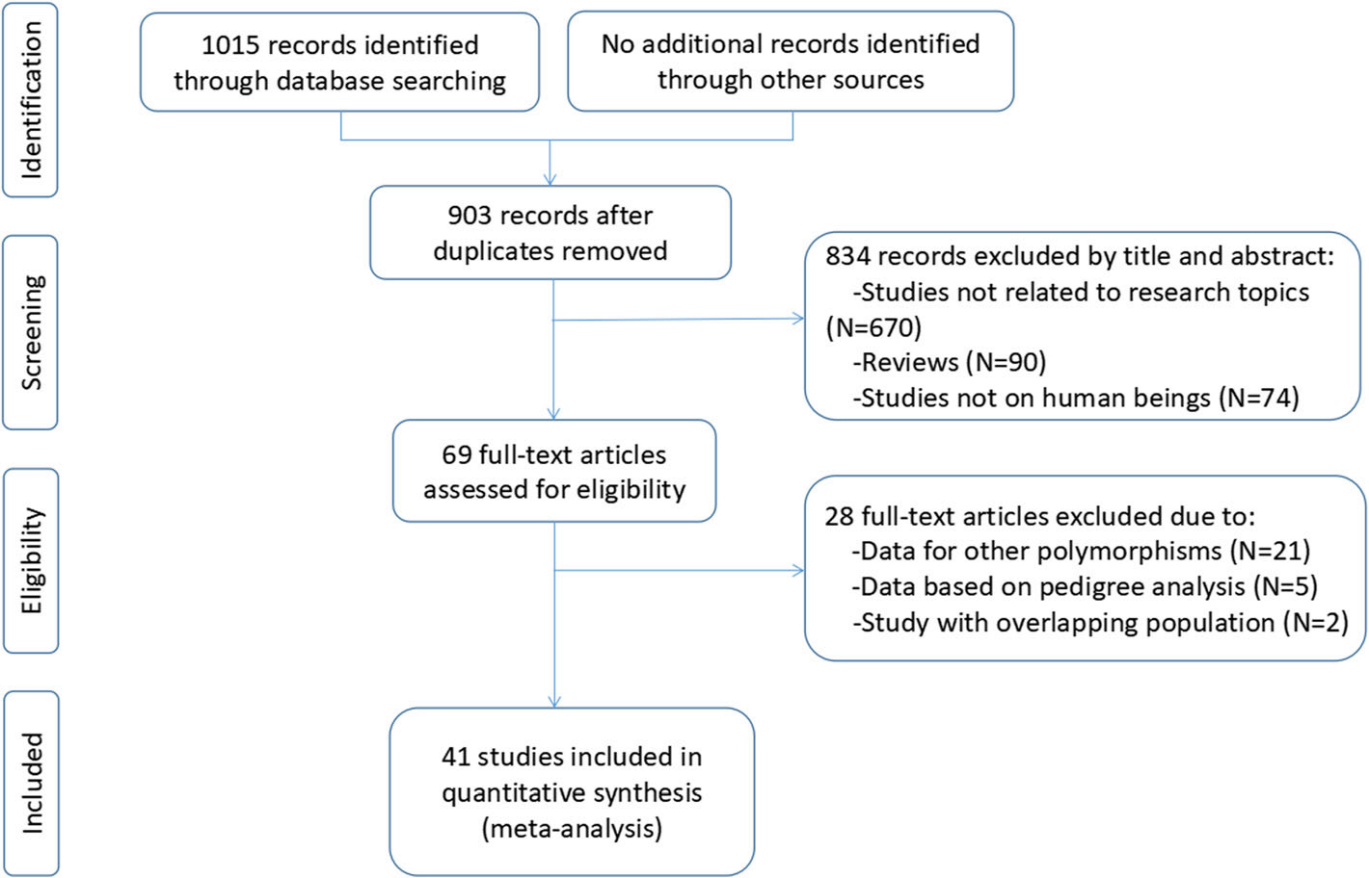
Flow diagram of the study selection process

Initial search of the databases yielded 903 articles. Eight hundred thirty-four studies were excluded according to the titles and abstracts. Then full-text articles were retrieved and assessed on the basis of the inclusion criteria. Twenty-eight studies were ineligible for the following reasons: 21 studies presented data for other polymorphisms, 5 studies were based on pedigree analysis, and 2 studies had subjects overlapping with other publications. In the end, 41 studies were selected for this meta-analysis (Fig. 1). Out of them, 26 studies (34 comparisons) involved Caucasian subjects; three studies (three comparisons) involved Asian subjects; and 12 studies (24 comparisons) had to do with the subjects of other ethnic origins. Eight of the included studies were case-control studies, while the remaining 33 studies were cohort studies.

The references for the studies included in the present meta-analysis are listed in Additional file 1. The characteristics of the studies included in the lipid association analysis are summarized in Additional file 2: Table S1. The plasma lipid levels by the genotypes of the rs854560 polymorphism are presented in Additional file 2: Table S2.

### Associations of the *PON1* rs854560 polymorphism with plasma lipid levels

**Fig. 2:**
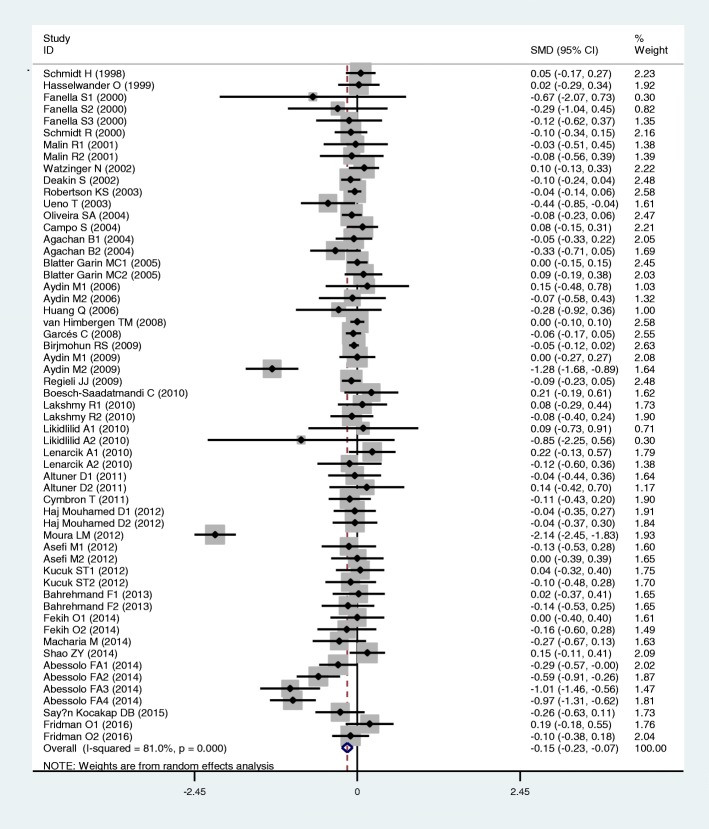
Forest plot of the meta-analysis between the *PON1* rs854560 polymorphism and plasma high-density lipoprotein cholesterol (HDL-C) levels

**Fig. 3:**
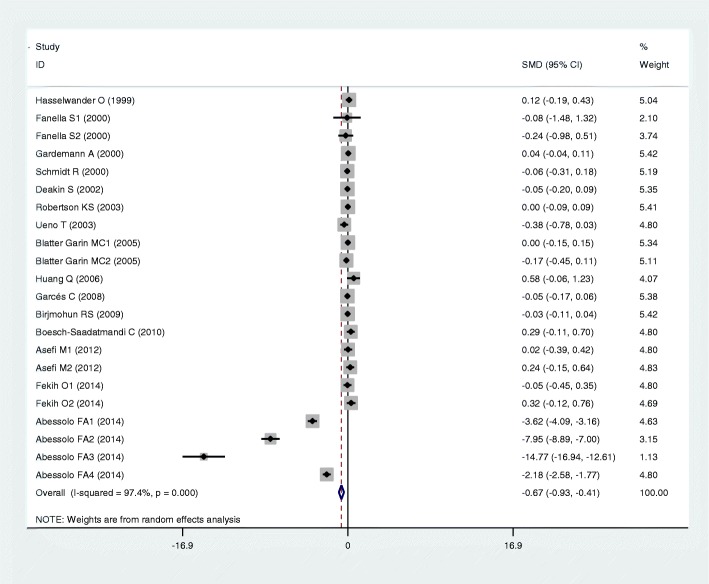
Forest plot of the meta-analysis between the *PON1* rs854560 polymorphism and plasma apolipoprotein A-I (APOA1) levels

**Fig. 4:**
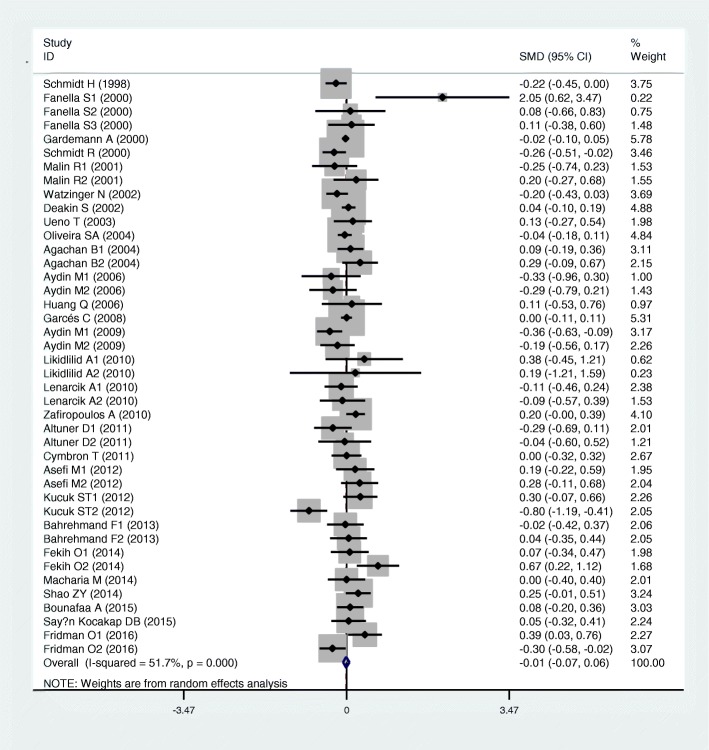
Forest plot of the meta-analysis between the *PON1* rs854560 polymorphism and triglycerides (TG) levels

**Fig. 5:**
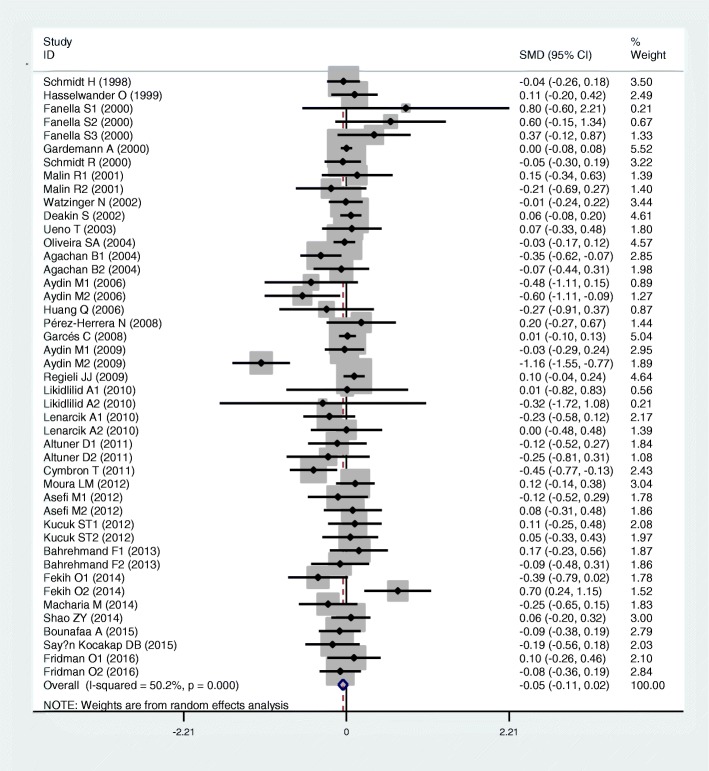
Forest plot of the meta-analysis between the *PON1* rs854560 polymorphism and plasma total cholesterol (TC) levels

**Fig. 6:**
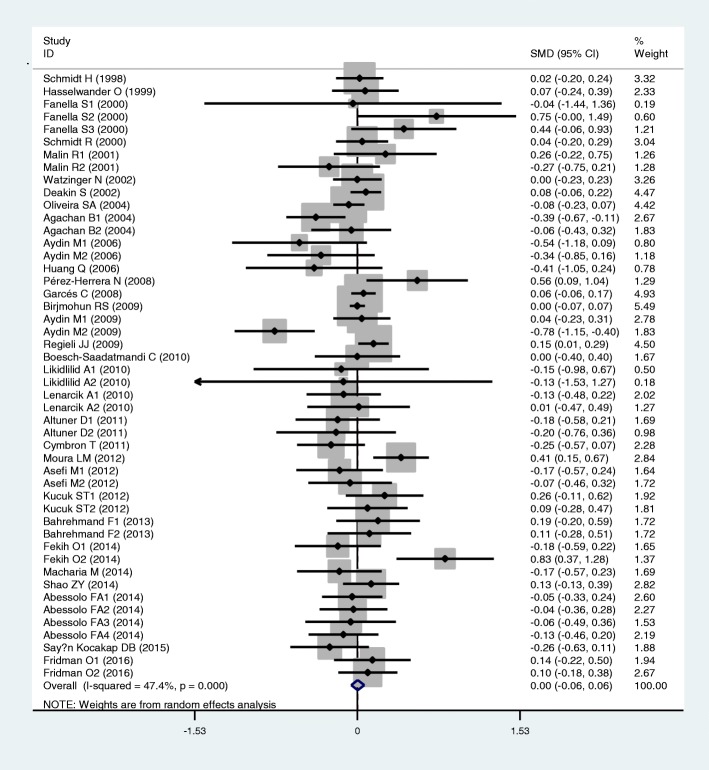
Forest plot of the meta-analysis between the *PON1* rs854560 polymorphism and plasma low-density lipoprotein cholesterol (LDL-C) levels

Results of the analyses on all comparisons showed that the M allele carriers had lower levels of HDL-C (SMD = − 0.15, 95% CI = − 0.23--0.07, *P* < 0.01) and APOA1 (SMD = − 0.67, 95% CI = − 0.93--0.41, P < 0.01) than the non-carriers (Table 1, Figs. 2 and 3). When the analyses were limited to the studies in HWE, the significant associations between the rs854560 polymorphism and plasma levels of HDL-C (SMD = − 0.04, 95% CI = − 0.07--0.01, *P* = 0.05) and APOA1 (SMD = − 0.74, 95% CI = − 1.03--0.46, *P* < 0.01) were also detected (Table 1). No associations were found between the rs854560 polymorphism and plasma levels of TG (SMD = − 0.01, 95% CI = − 0.07-0.06, *P* = 0.87), TC (SMD = − 0.05, 95% CI = − 0.11-0.02, *P* = 0.15), and LDL-C (SMD = 0.00, 95% CI = − 0.06-0.06, *P* = 0.91) (Table 1, Figs. 4, 5 and 6).

**Table 1:** Meta-analysis between the *PON1* rs854560 polymorphism and plasma lipid levels

Groups or subgroups	Comparisons (Subjects)	SMD (95% CI)	*P*_Heterogeneity_	*P*_SMD_
TG				
All	42 (12791)	− 0.01 (− 0.07–0.06)	< 0.01	0.87
HWE	33 (9805)	-0.02 (− 0.10–0.07)	< 0.01	0.71
Male	3 (2920)	-0.02 (− 0.09–0.05)	0.42	0.58
Caucasian	21 (8278)	-0.01 (− 0.09–0.07)	0.01	0.81
Asian	3 (956)	0.22 (0.01–0.41)	0.86	0.05
Other ethnic	18 (3557)	− 0.03 (− 0.18–0.11)	< 0.01	0.64
CHD	5 (802)	0.08 (− 0.20–0.35)	0.01	0.59
T2DM	3 (693)	0.38 (− 0.61–1.37)	0.01	0.45
Healthy or control	20 (3268)	−0.05 (− 0.19–0.08)	< 0.01	0.45
Case-control	6 (5934)	0.00 (−0.05–0.06)	0.45	0.90
TC				
All	45 (13715)	−0.05 (− 0.11–0.02)	< 0.01	0.15
HWE	35 (10411)	−0.01 (− 0.06–0.04)	0.29	0.68
Male	5 (3786)	0.02 (−0.04–0.09)	0.51	0.47
Caucasian	24 (9202)	0.01 (−0.03–0.05)	0.56	0.68
Asian	3 (956)	0.03 (−0.18–0.24)	0.63	0.79
Other ethnic	18 (3557)	−0.16 (− 0.33–0.01)	< 0.01	0.07
CHD	6 (1593)	0.04 (−0.06–0.14)	0.61	0.45
Healthy or control	20 (3305)	−0.11 (− 0.26–0.03)	< 0.01	0.12
Case-control	7 (6252)	0.01 (−0.05–0.07)	0.77	0.73
LDL-C				
All	48 (14319)	0.00 (−0.06–0.06)	< 0.01	0.91
HWE	35 (10632)	0.03 (−0.04–0.09)	0.03	0.39
Male	4 (1002)	0.17 (−0.09–0.43)	0.11	0.20
Caucasian	24 (9423)	0.06 (0.01–0.12)	0.19	0.03
Asian	5 (1187)	0.09 (−0.17–0.34)	0.32	0.49
Other ethnic	19 (3709)	−0.11 (− 0.23–0.01)	0.01	0.08
CHD	5 (1387)	0.09 (−0.04–0.23)	0.28	0.17
T2DM	4 (486)	−0.09 (− 0.34–0.15)	0.85	0.46
Healthy or control	24 (4919)	0.01 (−0.10–0.13)	< 0.01	0.80
Case-control	6 (6364)	0.05 (−0.06–0.17)	0.03	0.36
HDL-C				
All	57 (19354)	−0.15 (− 0.23--0.07)	< 0.01	< 0.01
HWE	41 (15157)	−0.04 (− 0.07--0.01)	0.99	0.05
Male	4 (2518)	−0.06 (− 0.14–0.02)	0.95	0.16
Female	3 (1714)	0.01 (−0.09–0.11)	0.42	0.81
Caucasian	41 (15269)	−0.11 (− 0.20--0.02)	< 0.01	0.02
Asian	6 (1405)	−0.13 (− 0.39–0.13)	0.18	0.33
Other ethnic	11 (2680)	−0.32 (− 0.52--0.13)	< 0.01	< 0.01
CHD	7 (2221)	−0.03 (− 0.12–0.06)	0.65	0.49
T2DM	4 (848)	−0.35 (− 0.68--0.02)	0.21	0.04
Healthy or control	29 (8424)	−0.11 (− 0.20--0.02)	< 0.01	0.02
Case-control	7 (6582)	−0.41 (− 0.77--0.04)	< 0.01	0.03
APOA1				
All	22 (13391)	−0.67 (− 0.93--0.41)	< 0.01	< 0.01
HWE	20 (12398)	−0.74 (−1.03--0.46)	< 0.01	< 0.01
Caucasian	14 (12212)	−0.01 (− 0.05–0.03)	0.78	0.66
Other ethnic	6 (808)	−4.46 (−6.69--2.24)	< 0.01	< 0.01
Healthy or control	9 (4375)	−0.13 (− 0.42–0.16)	< 0.01	0.39
Case-control	4 (6891)	−0.02 (− 0.10–0.05)	0.15	0.56

*SMD* standardized mean difference, *95% CI* 95% confidence interval, *HWE* Hardy-Weinberg equilibrium, *TG* triglyceride, *TC* total cholesterol, *LDL-C* low-density lipoprotein cholesterol, *HDL-C* high-density lipoprotein cholesterol, *APOA1* apolipoprotein A-I, *CHD* coronary heart disease, *T2DM* type 2 diabetes mellitus

From the subgroup analyses stratified by the characteristics of the subjects, significant associations of the rs854560 polymorphism with lower levels of HDL-C (SMD = − 0.11, 95% CI = − 0.20--0.02, *P* = 0.02) and higher levels of LDL-C (SMD = 0.06, 95% CI = 0.01–0.12, *P* = 0.03) were detected in Caucasians. The rs854560 polymorphism was significantly associated with lower levels of HDL-C (SMD = − 0.32, 95% CI = − 0.52--0.13, *P* < 0.01) and APOA1 (SMD = − 4.46, 95% CI = − 6.69--2.24, *P* < 0.01) in the subjects of other ethnic origins. The rs854560 polymorphism was also significantly associated with higher levels of TG (SMD = 0.22, 95% CI = 0.01–0.41, *P* = 0.05) in Asians. When health status was taken into account, the significant association of the rs854560 polymorphism with lower levels of HDL-C was detected in the T2DM patients, the healthy/control subjects and the case-control subjects (Table 1).

### Evaluation of heterogeneity

The heterogeneity among studies was significant for the five lipid variables (TG: *I*^*2*^ = 51.7%, *P*_*heterogeneity*_ < 0.01; TC: *I*^*2*^ = 50.2%, *P*_*heterogeneity*_ < 0.01; LDL-C: *I*^*2*^ = 47.4%, *P*_*heterogeneity*_ < 0.01; HDL-C: *I*^*2*^ = 81.0%, *P*_*heterogeneity*_ < 0.01; APOA1: *I*^*2*^ = 97.4%, *P*_*heterogeneity*_ < 0.01). Six comparisons (Fanella S1 2000, Fekih O2 2014, Fridman O1 2016, Kucuk ST2 2012, Aydin M1 2009, Zafiropoulos A2 2010), 5 comparisons (Fekih O2 2014, Aydin M2 2009, Cymbron T 2011, Agachan B1 2004, Aydin M2 2006), 5 comparisons (Fekih O2 2014, Moura LM 2012, Pérez-Herrera N 2008, Aydin M2 2009, Agachan B1 2004), 4 comparisons (Moura LM 2012, Aydin M2 2009, Abessolo FA3 2014, Abessolo FA4 2014) and 4 comparisons (Abessolo FA1 2014, Abessolo FA2 2014, Abessolo FA3 2014, Abessolo FA4 2014) were identified as the main contributors to the heterogeneity for TG, TC, LDL-C, HDL-C and APOA1, respectively, by using Galbraith plots. The SMD values and 95% CIs of TG (SMD = − 0.02, 95% CI = − 0.06-0.02, *P*_*Heterogeneity*_ = 0.38, *P*_*SMD*_ = 0.40), TC (SMD = − 0.00, 95% CI = − 0.04-0.04, *P*_*Heterogeneity*_ = 0.69, *P*_*SMD*_ = 0.87), LDL-C (SMD = 0.01, 95% CI = − 0.03-0.05, *P*_*Heterogeneity*_ = 0.65, *P*_*SMD*_ = 0.51) and HDL-C (SMD = − 0.05, 95% CI = − 0.08--0.02, *P*_*heterogeneity*_ = 0.83, *P*_*SMD*_ < 0.01) did not change substantially after excluding these outlier comparisons. However, SMD value and 95% CI of APOA1 (SMD = − 0.01, 95% CI = − 0.04-0.03, *P*_*heterogeneity*_ = 0.42, *P*_*SMD*_ = 0.69) changed significantly after excluding the outlier comparisons.

### Publication bias test

The Begg’s funnel plot and Egger’s test were used to evaluate the publication bias in the literature. In the present study, Begg’s funnel plot showed no publication bias, and this was confirmed by Egger’s test (*P* = 0.43 for TG, 0.57 for TC, 0.56 for LDL-C, 0.98 for HDL-C, and 0.16 for APOA1).

## Discussion

To the best of our knowledge, this is the first time that the associations of the *PON1* rs854560 polymorphism with serum lipid levels are investigated. The present meta-analysis suggests that M allele of the rs854560 polymorphism is associated with lower levels of HDL-C and APOA1 in the total population. A number of case-control studies [7–9] demonstrated that M allele of the rs854560 polymorphism had a promoting role for CHD risk. In combination with our findings, it is possible that the association of rs854560 polymorphism with a higher risk of CHD is mediated by the decreased levels of HDL-C and APOA1 caused by M allele of the rs854560 polymorphism.

Subgroup analyses by ethnicity, gender and health condition were performed since they might be important variables in determining associative risk with lipid levels. For example, the present meta-analysis indicated that ethnicity might modulate the associations of the rs854560 polymorphism with HDL-C levels since the strong significant associations only existed in Caucasians (Table 1). A recent meta-analysis [23] revealed that M carriers of the rs854560 polymorphism had a higher risk of CHD than the non-carriers in the populations involved Caucasians. In combination with our findings, it is possible that the association between M allele of the rs854560 polymorphism and a higher risk of CHD in Caucasians was mediated by decreased HDL-C and APOA1 levels.

The *PON1* rs854560 polymorphism may result in markedly reduced levels of HDL-C and APOA1 by affecting PON1 activity [11]. In 2014, Abessolo et al. performed a study on T2DM patients to assess the relationships between rs854560 polymorphism, PON1 activity and plasma lipid levels [16]. The results showed that the *PON1* rs854560 polymorphism was significantly associated with decreased levels of HDL-C and APOA1 possibly via decreased serum PON1 activity. One possible explanation for this association is that the reduced PON1 enzyme activity might reduce the capacity of PON1-mediated inhibition of LDL oxidation [24, 25], which leads to increased levels of plasma ox-LDL (oxidized low-density lipoprotein) [26] and decreased levels of plasma HDL-C [27] and APOA1 [28]. This may also explain our findings.

Smoking is an established risk factor for CHD as it increases oxidative stress in the development of atherosclerosis. By using logistic regression analysis, Watzinger et al. found that smoking was independently associated with CHD [29]. While Robertson et al. reported that the association between smoking and CHD risk was significantly modified by the rs854560polymorphism [30]. In 2008, Van et al. conducted a study on a middle-aged woman and found that PON1 activity was associated with CHD risk and the risk could be modified by smoking [31]. These results revealed that the *PON1* rs854560 polymorphism might play an important role in predisposing the subjects to smoking-induced oxidative damage. However, more studies need to be performed to investigate the interaction between the *PON1* rs854560 polymorphism and smoking.

Some limitations to the present meta-analysis should be noted. First, dyslipidemia is involved with a large number of genes. However, the interactions of the *PON1* rs854560 polymorphism with other polymorphic loci on plasma lipid levels have not been investigated in this analysis due to lack of the original data from the included studies. Secondly, this meta-analysis only included the studies published in English and Chinese as it was very difficult to get full papers published in other languages.

## Conclusions

The meta-analysis suggests that the *PON1* rs854560 polymorphism is associated with a lower HDL-C level in Caucasians and the subjects of other ethnic origins. Further investigations of potential gene-gene and gene-environmental interactions are needed.

## Additional files

Additional file 1: The reference list for the studies included in the present meta-analysis. (DOC 45 kb)

Additional file 2: **Table S1**. Characteristics of the individual studies included in the meta-analysis between the *PON1* rs854560 polymorphism and plasma lipid levels; **Table S2**. Plasma lipid levels by the genotypes of the *PON1* rs854560 polymorphism. (DOC 387 kb)

## Abbreviations

95% CI: 95% confidence interval
APOA1: apolipoprotein A-I
CHD: coronary heart disease
HDL-C: high-density lipoprotein cholesterol
HWE: Hardy-Weinberg equilibrium
LDL-C: low-density lipoprotein cholesterol
PON1: paraoxonase 1
RCT: reverse cholesterol transport
SMD: standardized mean difference
T2DM: type 2 diabetes mellitus
TC: total cholesterol
TG: triglyceride
