# Supplementary material for: Associations of the PON1 rs854560 polymorphism with plasma lipid levels: a meta-analysis
Source: Lipids Health Dis. 2018 Dec 3;17:274. doi: 10.1186/s12944-018-0924-0 (PMC6278118; doi:10.1186/s12944-018-0924-0)
Supplement: Supplementary file 1 — The reference list for the studies included in the present meta-analysis. (DOC 45 kb) [file 12944_2018_924_MOESM1_ESM.doc]

**The reference list for the studies included in the present meta-analysis**

**R1.** Schmidt H, Schmidt R, Niederkorn K, Gradert A, Schumacher M, Watzinger N, Hartung HP, Kostner GM. Paraoxonase PON1 polymorphism leu-Met54 is associated with carotid atherosclerosis: results of the Austrian Stroke Prevention Study. Stroke. 1998; 29(10): 2043-8.

**R2.** Hasselwander O, Savage DA, McMaster D, Loughrey CM, McNamee PT, Middleton D, Nicholls DP, Maxwell AP, Young IS. Paraoxonase polymorphisms are not associated with cardiovascular risk in renal transplant recipients. Kidney Int. 1999; 56(1): 289-98.

**R3.** Fanella S, Harris SB, Young TK, Hanley AJ, Zinman B, Connelly PW, Hegele RA. Association between PON1 L/M55 polymorphism and plasma lipoproteins in two Canadian aboriginal populations. Clin Chem Lab Med. 2000; 38(5): 413-20.

**R4**. Gardemann A, Philipp M, Hess K, Katz N, Tillmanns H, Haberbosch W. The paraoxonase Leu-Met54 and Gln-Arg191 gene polymorphisms are not associated with the risk of coronary heart disease. Atherosclerosis. 2000; 152(2): 421-31.

**R5.** Schmidt R, Schmidt H, Fazekas F, Kapeller P, Roob G, Lechner A, Kostner GM, Hartung HP. MRI cerebral white matter lesions and paraoxonase PON1 polymorphisms: three-year follow-up of the austrian stroke prevention study. Arterioscler Thromb Vasc Biol. 2000; 20(7): 1811-6.

**R6.** Malin R, Lehtinen S, Luoma P, Näyhä S, Hassi J, Koivula T, Lehtimäki T. Serum lipid levels and M/L55 allele distribution of HDL paraoxonase gene in Saami and Finnish men. Int J Circumpolar Health. 2001; 60(1): 16-24.

**R7.** Watzinger N, Schmidt H, Schumacher M, Schmidt R, Eber B, Fruhwald FM, Zweiker R, Kostner GM, Klein W. Human paraoxonase 1 gene polymorphisms and the risk of coronary heart disease: a community-based study. Cardiology. 2002; 98(3): 116-22.

**R8.** Deakin S, Leviev I, Nicaud V, Brulhart Meynet MC, Tiret L, James RW; European Atherosclerosis Risk StudyGroup.Paraoxonase-1 L55M polymorphism is associated with an abnormal oral glucose tolerance test and differentiates high risk coronary disease families. J Clin Endocrinol Metab. 2002; 87(3): 1268-73.

**R9.** Robertson KS, Hawe E, Miller GJ, Talmud PJ, Humphries SE; Northwick Park Heart Study II. Human paraoxonase gene cluster polymorphisms as predictors of coronary heart disease risk in the prospective Northwick Park Heart Study II. Biochim Biophys Acta. 2003; 1639(3): 203-12.

**R10.** Ueno T, Shimazaki E, Matsumoto T, Watanabe H, Tsunemi A, Takahashi Y, Mori M, Hamano R, Fujioka T, Soma M, Matsumoto K, Kanmatsuse K. Paraoxonase1 polymorphism Leu-Met55 is associated with cerebral infarction in Japanesepopulation. Med Sci Monit. 2003; 9(6): CR208-12.

**R11.** Oliveira SA, Mansur AP, Ribeiro CC, Ramires JA, Annichino-Bizzacchi JM. PON1 M/L55 mutation protects high-risk patients against coronary artery disease. Int J Cardiol. 2004; 94(1): 73-7.

**R12.** Campo S, Sardo MA, Trimarchi G, Bonaiuto M, Fontana L, Castaldo M, Bonaiuto A, Saitta C, Bitto A, Manduca B, Riggio S, Saitta A. Association between serum paraoxonase (PON1) gene promoter T(-107)C polymorphism, PON1 activity and HDL levels in healthy Sicilian octogenarians. Exp Gerontol. 2004; 39(7): 1089-94.

**R13.** Agachan B, Yilmaz H, Karaali Z, Isbir T. Paraoxonase 55 and 192 polymorphism and its relationship to serum paraoxonase activity and serum lipids in Turkish patients with non-insulin dependent diabetes mellitus. Cell Biochem Funct. 2004; 22(3): 163-8.

**R14.** Blatter Garin MC, Moren X, James RW. Paraoxonase-1 and serum concentrations of HDL-cholesterol and apoA-I. J Lipid Res. 2006; 47(3): 515-20.

**R15.** Aydin M, Gencer M, Cetinkaya Y, Ozkok E, Ozbek Z, Kilic G, Orken C, Tireli H, Kara I. PON1 55/192 polymorphism, oxidative stress, type, prognosis and severity of stroke. IUBMB Life. 2006; 58(3): 165-72.

**R16.** Huang Q, Liu YH, Yang QD, Xiao B, Ge L, Zhang N, Xia J, Zhang L, Liu ZJ. Humann serum paraoxonase gene polymorphisms, Q192R and L55M, are not associated with the risk of cerebral infarction in Chinese Han population. Neurol Res. 2006; 28(5): 549-54.

**R17.** van Himbergen TM, van der Schouw YT, Voorbij HA, van Tits LJ, Stalenhoef AF, Peeters PH, Roest M. van Himbergen TM, van der Schouw YT, Voorbij HA, van Tits LJ, Stalenhoef AF, Peeters PH, Roest M. Paraoxonase (PON1) and the risk for coronary heart disease and myocardial infarction in a general population of Dutch women. Atherosclerosis. 2008; 199(2): 408-14.

**R18.** Pérez-Herrera N, May-Pech C, Hernández-Ochoa I, Castro-Mañé J, Rojas-García E, Borja-Aburto VH, Castillo-Burguete T, Quintanilla-Vega B. PON1Q192R polymorphism is associated with lipid profile in Mexican men with Mayanascendancy. Exp Mol Pathol. 2008; 85(2): 129-34.

**R19.** Garcés C, López-Simón L, Rubio R, Benavente M, Cano B, Ortega H, de Oya M. High-density lipoprotein cholesterol and paraoxonase 1 (PON1) genetics and serum PON1 activity in prepubertal children in Spain. Clin Chem Lab Med. 2008; 46(6): 809-13.

**R20.** Birjmohun RS, Vergeer M, Stroes ES, Sandhu MS, Ricketts SL, Tanck MW, Wareham NJ, Jukema JW, Kastelein JJ, Khaw KT, Boekholdt SM. Both paraoxonase-1 genotype and activity do not predict the risk of future coronary artery disease; the EPIC-Norfolk Prospective Population Study. PLoS One. 2009; 4(8): e6809.

**R21.** Aydin M, Gokkusu C, Ozkok E, Tulubas F, Unlucerci Y, Pamukcu B, Ozbek Z, Umman B. Association of genetic variants in Methylenetetrahydrofolate Reductase and Paraoxonase-1genes with homocysteine, folate and vitamin B12 in coronary artery disease. Mol Cell Biochem. 2009; 325(1-2): 199-208.

**R22.** Regieli JJ, Jukema JW, Doevendans PA, Zwinderman AH, Kastelein JJ, Grobbee DE, van der Graaf Y. Paraoxonase variants relate to 10-year risk in coronary artery disease: impact of a high-density lipoprotein-bound antioxidant in secondary prevention. J Am Coll Cardiol. 2009; 54(14): 1238-45.

**R23.** Boesch-Saadatmandi C, Rimbach G, Schrader C, Kofler BM, Armah CK, Minihane AM. Determinants of paraoxonase activity in healthy adults. Mol Nutr Food Res. 2010; 54(12): 1842-50.

**R24.** Lakshmy R, Ahmad D, Abraham RA, Sharma M, Vemparala K, Das S, Reddy KS, Prabhakaran D. Paraoxonase gene Q192R & L55M polymorphisms in Indians with acute myocardial infarction &association with oxidized low density lipoprotein. Indian J Med Res. 2010; 131: 522-9.

**R25.** [Likidlilid A](https://www.ncbi.nlm.nih.gov/pubmed/?term=Likidlilid A%5BAuthor%5D&cauthor=true&cauthor_uid=21302675), [Akrawinthawong K](https://www.ncbi.nlm.nih.gov/pubmed/?term=Akrawinthawong K%5BAuthor%5D&cauthor=true&cauthor_uid=21302675), [Poldee S](https://www.ncbi.nlm.nih.gov/pubmed/?term=Poldee S%5BAuthor%5D&cauthor=true&cauthor_uid=21302675), [Sriratanasathavorn C](https://www.ncbi.nlm.nih.gov/pubmed/?term=Sriratanasathavorn C%5BAuthor%5D&cauthor=true&cauthor_uid=21302675).Paraoxonase 1 polymorphisms as the risk factor of coronary heart disease in a Thai population.[Acta Cardiol.](https://www.ncbi.nlm.nih.gov/pubmed/21302675) 2010;65(6):681-91.

**R26.** Lenarcik A, Bidzińska-Speichert B, Tworowska-Bardzińska U. The role of chronic inflammation and Leu55Met PON1 polymorphism in the pathogenesis of polycystic ovary syndrome. Gynecol Endocrinol. 2010; 26(9): 673-83.

**R27.** Zafiropoulos A, Linardakis M, Jansen EH, Tsatsakis AM, Kafatos A, Tzanakakis GN. Paraoxonase 1 R/Q alleles are associated with differential accumulation of saturated versus20:5n3 fatty acid in human adipose tissue. J Lipid Res. 2010; 51(7): 1991-2000.

**R28.** Altuner D, Ates I, Suzen SH, Koc GV, Aral Y, Karakaya A. The relationship of PON1 QR 192 and LM 55 polymorphisms with serum paraoxonase activities of Turkish diabetic patients. Toxicol Ind Health. 2011; 27(10): 873-8.

**R29.** Cymbron T, Raposo M, Kazachkova N, Bettencourt C, Silva F, Santos C, Dahmani Y, Lourenço P, Ferin R, Pavão ML, Lima M. Cross-sectional study of risk factors for atherosclerosis in the Azorean population. Ann Hum Biol. 2011; 38(3): 354-9.

**R30.** Haj Mouhamed D, Ezzaher A, Mechri A, Neffati F, Omezzine A, Bouslama A, Gaha L, Douki W, Najjar MF. Effect of cigarette smoking on paraoxonase 1 activity according to PON1 L55M and PON1 Q192R gene polymorphisms. Environ Health Prev Med. 2012; 17(4): 316-21.

**R31.** Moura LM, Faria S, Brito M, Pinto FJ, Kristensen SD, Barros IM, Rajamannan N, Rocha-Gonçalves F. Relationship of PON1 192 and 55 gene polymorphisms to calcific valvular aortic stenosis. Am J Cardiovasc Dis. 2012; 2(2): 123-32.

**R32.** Asefi M, Vaisi-Raygani A, Bahrehmand F, Kiani A, Rahimi Z, Nomani H, Ebrahimi A, Tavilani H, Pourmotabbed T. Paraoxonase 1 (PON1) 55 polymorphism, lipid profiles and psoriasis. Br J Dermatol. 2012; 167(6): 1279-86.

**R33.** Kucuk ST, Ademoglu E, Turkoglu UM, Bilge AK. Distribution of PON L/M55 and Q/R192 Genotypes in Turkish Patients with Angiographically-Defined Coronary Artery Disease: Effects on Serum Lipids. Turkiye Klinikleri Journal of Medical Sciences. 2013; 33(3): 769-776.

**R34.** Bahrehmand F, Vaisi-Raygani A, Ahmadi R, Kiani A, Rahimi Z, Tavilani H, Pourmotabbed T. Paraoxonase (PON1) 55 polymorphism and association with systemic lupus erythematosus. Iran J Allergy Asthma Immunol. 2013; 12(3): 211-9.

**R35.** Fekih O, Triki S, Hellara I, Neffati F, Rejeb J, Ommezzine A, Chouchane S, Guediche MN, Bouslama A, Najjar MF. Can paraoxonase 1 polymorphisms (L55 M and Q192 R) protect children with type 1 diabetes against lipid abnormalities? J Clin Lipidol. 2014; 8(3): 249-55.

**R36.** Macharia M, Kengne AP, Blackhurst DM, Erasmus RT, Matsha TE. Paraoxonase1 genetic polymorphisms in a mixed ancestry African population. Mediators Inflamm. 2014; 2014: 217019.

**R37.** Shao ZY, Li JR, Wang XD. Analysis of paraoxonase 1 gene polymorphisms in type 2 diabetic patients with coronary artery disease. Chin J Cardiovasc Med. 2014; 19(6): 426-429.

**R38.** Abessolo FA, Bruno MJ, N'Negue MA, Yangou M, Ngoumilama E. Enzymatic and genetic polymorphisms of paraoxonase-1 in the Gabonese population: the relation to lipid parameters in patients with diabetes. Journal of Endocrinology Metabolism & Diabetes. 2012; 17 (2): 92-99.

**R39.** Bounafaa A, Berrougui H, Ghalim N, Nasser B, Bagri A, Moujahid A, Ikhlef S, Camponova P, Yamoul N, Simo OK, Essamadi A, Khalil A. Association between Paraoxonase 1 (PON1) Polymorphisms and the Risk of Acute coronary syndrome in a North African Population. PLoS One. 2015; 10(8): e0133719.

**R40.** Sayın Kocakap DB, Doğru MT, Şimşek V, Çabuk F, Yıldırım N, Çelik Y, Alyılmaz Bekmez S, Erdem S. The association of paraoxonase 1 gene L55M polymorphism with the extent and severity of coronary artery disease in the Turkish population and its dependence on gender. Anatol J Cardiol. 2016; 16(3): 175-82.

**R41.** Fridman O, Gariglio L, Riviere S, Porcile R, Fuchs A, Potenzoni M. Paraoxonase 1 gene polymorphisms and enzyme activities in coronary artery disease and its relationship to serum lipids and glycemia. Arch Cardiol Mex. 2016; 86(4): 350-357.
