# Supplementary material for: Associations of the PON1 rs854560 polymorphism with plasma lipid levels: a meta-analysis
Source: Lipids Health Dis. 2018 Dec 3;17:274. doi: 10.1186/s12944-018-0924-0 (PMC6278118; doi:10.1186/s12944-018-0924-0)
Supplement: Supplementary file 2 — Table S1. Characteristics of the individual studies included in the meta-analysis between the PON1 rs854560 polymorphism and plasma lipid levels; Table S2. Plasma lipid levels by the genotypes of the PON1 rs854560 polymorphism. (DOC 387 kb) [file 12944_2018_924_MOESM2_ESM.doc]

**Table S1.** Characteristics of the individual studies included in the meta-analysis between the *PON1* rs854560 polymorphism and plasma lipid levels

**Table S2:** Plasma lipid levels by the genotypes of the *PON1* rs854560 polymorphism.

**Table S1 : Characteristics of the individual studies included in the meta-analysis between the *PON1* rs854560 polymorphism and plasma lipid levels**

| **First author, reference** | **year** | **Ethnicity** | **Gender** | **Study population** | **Outcomes** |
| --- | --- | --- | --- | --- | --- |
| Schmidt H [R1] | 1998 | Caucasian | M/F | Healthy subjects | TG, TC, LDL-C, HDL-C |
| Hasselwander O [R2] | 1999 | Caucasian | M/F | Renal transplant recipients | TC, LDL-C, HDL-C, APOA1 |
| Fanella S1 [R3] | 2000 | Caucasian | M/F | T2DM patients | TG, TC, LDL-C, HDL-C, APOA1 |
| Fanella S2 [R3] | 2000 | Caucasian | M/F | Patients without T2DM | TG, TC, LDL-C, HDL-C, APOA1 |
| Fanella S3 [R3] | 2000 | Caucasian | M/F | Healthy subjects | TG, TC, LDL-C, HDL-C |
| Gardemann A [R4] | 2000 | Caucasian | M | CHD patients and control subjects | TG, TC, APOA1 |
| Schmidt R [R5] | 2000 | Caucasian | M/F | Healthy subjects | TG, TC, LDL-C, HDL-C, APOA1 |
| Malin R1 [R6] | 2001 | Caucasian | M | Healthy subjects | TG, TC, LDL-C, HDL-C |
| Malin R2 [R6] | 2001 | Caucasian | M | Healthy subjects | TG, TC, LDL-C, HDL-C |
| Watzinger N [R7] | 2002 | Caucasian | M/F | Healthy subjects | TG, TC, LDL-C, HDL-C |
| Deakin S [R8] | 2002 | Caucasian | M/F | CHD high-risk subjects and control subjects | TG, TC, LDL-C, HDL-C, APOA1 |
| Robertson KS [R9] | 2003 | Caucasian | M | Healthy subjects | HDL-C, APOA1 |
| Ueno T [R10] | 2003 | Asian | M/F | Cerebral infarction patients and control subjects | TG, TC, HDL-C, APOA1 |
| Oliveira SA [R11] | 2004 | Other ethnic | M/F | CHD patients and control subjects | TG, TC, LDL-C, HDL-C |
| Campo S [R12] | 2004 | Caucasian | M/F | Healthy subjects | HDL-C |
| Agachan B1 [R13] | 2004 | Other ethnic | M/F | Noninsulin dependent diabetes mellitus patients | TG, TC, LDL-C, HDL-C |
| Agachan B2 [R13] | 2004 | Other ethnic | M/F | Control subjects | TG, TC, LDL-C, HDL-C |
| Blatter Garin MC1 [R14] | 2005 | Caucasian | M/F | CHD patients | HDL-C, APOA1 |
| Blatter Garin MC2 [R14] | 2005 | Caucasian | M/F | Control subjects | HDL-C, APOA1 |
| Aydin M1 [R15] | 2006 | Other ethnic | M/F | Stroke patients | TG, TC, LDL-C, HDL-C |
| Aydin M2 [R15] | 2006 | Other ethnic | M/F | Control subjects | TG, TC, LDL-C, HDL-C |
| Huang Q [R16] | 2006 | Asian | M/F | Healthy subjects | TG, TC, LDL-C, HDL-C, APOA1 |
| van Himbergen TM [R17] | 2008 | Caucasian | F | Healthy subjects | HDL-C |
| Pérez-Herrera N [R18] | 2008 | Caucasian | M | Healthy subjects | TC, LDL-C |
| Garcés C [R19] | 2008 | Caucasian | M/F | Healthy subjects | TG, TC, LDL-C, HDL-C, APOA1 |
| Birjmohun RS [R20] | 2009 | Caucasian | M/F | CHD patients and control subjects | LDL-C, HDL-C, APOA1 |
| Aydin M1 [R21] | 2009 | Other ethnic | M/F | CHD patients | TG, TC, LDL-C, HDL-C |
| Aydin M2 [R21] | 2009 | Other ethnic | M/F | Control subjects | TG, TC, LDL-C, HDL-C |
| Regieli JJ [R22] | 2009 | Caucasian | M | CHD patients | TC, LDL-C, HDL-C |
| Boesch-Saadatmandi C [R23] | 2010 | Caucasian | M/F | Healthy subjects | LDL-C, HDL-C, APOA1 |
| Lakshmy R1 [R24] | 2010 | Caucasian | M/F | CHD patients | HDL-C |
| Lakshmy R2 [R24] | 2010 | Caucasian | M/F | Control subjects | HDL-C |
| Likidlilid A1 [R25] | 2010 | Other ethnic | M/F | Hyperlipidemia patients | TG, TC, LDL-C, HDL-C |
| Likidlilid A2 [R25] | 2010 | Other ethnic | M/F | Control subjects | TG, TC, LDL-C, HDL-C |
| Lenarcik A1 [R26] | 2010 | Caucasian | F | Polycystic ovary syndrome patients | TG, TC, LDL-C, HDL-C |
| Lenarcik A2 [R26] | 2010 | Caucasian | F | Control subjects | TG, TC, LDL-C, HDL-C |
| Zafiropoulos A [R27] | 2010 | Caucasian | M/F | Healthy subjects | TG |
| Altuner D1 [R28] | 2011 | Other ethnic | M/F | T2DM patients | TG, TC, LDL-C, HDL-C |
| Altuner D2 [R28] | 2011 | Other ethnic | M/F | Control subjects | TG, TC, LDL-C, HDL-C |
| Cymbron T [R29] | 2011 | Caucasian | M/F | Healthy subjects | TG, TC, LDL-C, HDL-C |
| Haj Mouhamed D1 [R30] | 2012 | Other ethnic | M/F | Healthy subjects | HDL-C |
| Haj Mouhamed D2 [R30] | 2012 | Other ethnic | M/F | Healthy subjects | HDL-C |
| Moura LM [R31] | 2012 | Caucasian | M/F | CAVS patients and control subjects | TC, LDL-C, HDL-C |
| Asefi M1 [R32] | 2012 | Caucasian | M/F | Psoriasis patients | TG, TC, LDL-C, HDL-C, APOA1 |
| Asefi M2 [R32] | 2012 | Caucasian | M/F | Control subjects | TG, TC, LDL-C, HDL-C, APOA1 |
| Kucuk ST [R33] | 2012 | Other ethnic | M/F | CHD patients | TG, TC, LDL-C, HDL-C |
| Kucuk ST [R33] | 2012 | Other ethnic | M/F | Control subjects | TG, TC, LDL-C, HDL-C |
| Bahrehmand F1 [R34] | 2013 | Caucasian | M/F | Systemic lupus erythematosus patients | TG, TC, LDL-C, HDL-C |
| Bahrehmand F2 [R34] | 2013 | Caucasian | M/F | Control subjects | TG, TC, LDL-C, HDL-C |
| Fekih O1 [R35] | 2014 | Other ethnic | M/F | T1DM patients | TG, TC, LDL-C, HDL-C, APOA1 |
| Fekih O2 [R35] | 2014 | Other ethnic | M/F | Control subjects | TG, TC, LDL-C, HDL-C, APOA1 |
| Macharia M [R36] | 2014 | Other ethnic | M/F | Hypertension patients and control subjects | TG, TC, LDL-C, HDL-C |
| Shao ZY [R37] | 2014 | Asian | M/F | DM-CAD or DM and control subjects | TG, TC, LDL-C, HDL-C |
| Abessolo FA1 [R38] | 2014 | Other ethnic | M/F | Hypertension patients | LDL-C, HDL-C, APOA1 |
| Abessolo FA2 [R38] | 2014 | Other ethnic | M/F | T2DM patients | LDL-C, HDL-C, APOA1 |
| Abessolo FA3 [R38] | 2014 | Other ethnic | M/F | Hypertension+T2DM patients | LDL-C, HDL-C, APOA1 |
| Abessolo FA4 [R38] | 2014 | Other ethnic | M/F | Control subjects | LDL-C, HDL-C, APOA1 |
| Bounafaa A [R39] | 2015 | Caucasian | M/F | Acute coronary syndrome patients | TG, TC |
| Sayın Kocakap DB [R40] | 2015 | Other ethnic | M/F | Stable angina patients | TG, TC, LDL-C, HDL-C |
| Fridman O1 [R41] | 2016 | Caucasian | M/F | CHD patients | TG, TC, LDL-C, HDL-C |
| Fridman O2 [R41] | 2016 | Caucasian | M/F | Control subjects | TG, TC, LDL-C, HDL-C |

**Table S2:** Plasma lipid levels by the genotypes of the *PON1* rs854560 polymorphism.

| **First author, reference** | **Genotype** | |  | **TG, mmol/L** | |  | **TC, mmol/L** | |  | **LDL-C, mmol/L** | |  | **HDL-C, mmol/L** | |  | **APOA1,g/L** | |
| --- | --- | --- | --- | --- | --- | --- | --- | --- | --- | --- | --- | --- | --- | --- | --- | --- | --- |
|  | **LL** | **LM+MM** |  | **LL** | **LM+MM** |  | **LL** | **LM+MM** |  | **LL** | **LM+MM** |  | **LL** | **LM+MM** |  | **LL** | **LM+MM** |
| Schmidt H [R1] | 137 | 179 |  | 1.78±1.14 | 1.56±0.85 |  | 5.91± 1.01 | 5.87± 1.01 |  | 3.84±0.95 | 3.86±0.87 |  | 1.27±0.41 | 1.29±0.38 |  | - | - |
| Hasselwander O [R2] | 65 | 100 |  | - | - |  | 6.02±1.53 | 6.17±1.26 |  | 3.72±1.36 | 3.81±1.11 |  | 1.13±0.47 | 1.14±0.40 |  | 1.8±0.33 | 1.84±0.34 |
| Fanella S1 [R3] | 113 | 2 |  | 2.00±0.91 | 3.87±1.27 |  | 5.06±0.92 | 5.80±0.89 |  | 2.94±0.72 | 2.91±0.89 |  | 1.19±0.30 | 0.99±0.11 |  | 1.51±0.25 | 1.49±0.41 |
| Fanella S2 [R3] | 471 | 7 |  | 1.25±0.61 | 1.30±0.55 |  | 4.27±0.87 | 4.79±0.92 |  | 2.44±0.75 | 3.00±0.83 |  | 1.27±0.27 | 1.19±0.36 |  | 1.48±0.21 | 1.43±0.31 |
| Fanella S3 [R3] | 226 | 17 |  | 1.06±0.55 | 1.12±0.54 |  | 4.95±1.05 | 5.34±0.92 |  | 3.02±0.92 | 3.42±0.83 |  | 1.44±0.40 | 1.39±0.48 |  | - | - |
| Gardemann A [R4] | 1153 | 1631 |  | 1.72± 0.97 | 1.7±0.98 |  | 5.43±1.11 | 5.43±1.12 |  | - | - |  | - | - |  | 1.43±0.29 | 1.44±0.28 |
| Schmidt R [R5] | 111 | 153 |  | 1.83±1.61 | 1.52±0.72 |  | 6.05± 1.02 | 6.0±0.96 |  | 3.91±0.93 | 3.95±0.86 |  | 1.38±0.44 | 1.34±0.39 |  | 1.78±0.32 | 1.76±0.3 |
| Malin R1 [R6] | 29 | 39 |  | 1.56±1.36 | 1.3±0.68 |  | 6.47±1.84 | 6.69±1.2 |  | 4.47±1.62 | 4.82±1.06 |  | 1.28±0.37 | 1.27±0.37 |  | - | - |
| Malin R2 [R6] | 31 | 37 |  | 1.31±0.79 | 1.49±0.95 |  | 6.94±1.41 | 6.65±1.36 |  | 4.98±1.28 | 4.65±1.2 |  | 1.36±0.29 | 1.33±0.41 |  | - | - |
| Watzinger N [R7] | 131 | 172 |  | 1.73±0.97 | 1.55±0.84 |  | 5.86±1 | 5.85±0.98 |  | 3.83±0.95 | 3.83±0.86 |  | 1.25±0.4 | 1.29±0.39 |  | - | - |
| Deakin S [R8] | 309 | 466 |  | 0.97 ±0.53 | 0.99±0.4 |  | 4.39 ±0.88 | 4.44 ±0.8 |  | 2.75±0.7 | 2.81 ±0.78 |  | 1.20±0.18 | 1.18±0.21 |  | 1.01±0.18 | 1.00±0.2 |
| Robertson KS [R9] | 716 | 875 |  | - | - |  | - | - |  | - | - |  | 0.81±0.25 | 0.80±0.24 |  | 1.63±0.32 | 1.63±0.32 |
| Ueno T [R10] | 191 | 27 |  | 1.56±1.06 | 1.7±0.98 |  | 5.24±0.98 | 5.31±0.89 |  | - | - |  | 1.32±0.49 | 1.11±0.36 |  | 1.29±0.29 | 1.18±0.29 |
| Oliveira SA [R11] | 318 | 410 |  | 2.19±1.09 | 2.13±1.85 |  | 5.48±1.16 | 5.45±1.1 |  | 3.52±1.05 | 3.44±0.95 |  | 1.12±0.35 | 1.09±0.37 |  | - | - |
| Campo S [R12] | 128 | 172 |  | - | - |  | - | - |  | - | - |  | 1.43±0.24 | 1.45±0.26 |  | - | - |
| Agachan B1 [R13] | 111 | 94 |  | 1.73±0.86 | 1.82±1.22 |  | 5.17±1.25 | 4.72±1.35 |  | 3.16±1 | 2.76±1.05 |  | 1.08±0.34 | 1.06±0.43 |  | - | - |
| Agachan B2 [R13] | 51 | 58 |  | 1.38±0.52 | 1.56±0.7 |  | 4.28±0.75 | 4.22± 0.97 |  | 2.97±0.89 | 2.92±0.87 |  | 1.11±0.42 | 0.98±0.37 |  | - | - |
| Blatter Garin MC1 [R14] | 249 | 461 |  | - | - |  | - | - |  | - | - |  | 1.12±0.32 | 1.12±0.23 |  | 0.93±0.16 | 0.93±0.19 |
| Blatter Garin MC2 [R14] | 83 | 116 |  | - | - |  | - | - |  | - | - |  | 1.28±0.36 | 1.31±0.29 |  | 1.07±0.27 | 1.03±0.21 |
| Aydin M1 [R15] | 12 | 53 |  | 2.06±0.63 | 1.86±0.61 |  | 5.73±1.46 | 5.08±1.33 |  | 3.77±1.45 | 3.20±0.94 |  | 1.06±0.3 | 1.11±0.34 |  | - | - |
| Aydin M2 [R15] | 20 | 64 |  | 1.59±0.48 | 1.44±0.53 |  | 4.48±0.58 | 3.98±0.9 |  | 3.15±0.42 | 2.99±0.48 |  | 1.09±0.21 | 1.07±0.29 |  | - | - |
| Huang Q [R16] | 143 | 10 |  | 1.59 ± 0.96 | 1.70±0.94 |  | 4.14 ±0.91 | 3.89 ± 1.12 |  | 2.61± 0.78 | 2.29 ±0.89 |  | 1.27±0.88 | 1.03±0.14 |  | 1.07±0.19 | 1.18±0.18 |
| van Himbergen TM [R17] | 601 | 913 |  | - | - |  | - | - |  | - | - |  | 1.6±0.4 | 1.6±0.4 |  | - | - |
| Pérez-Herrera N [R18] | 47 | 28 |  | - | - |  | 4.28±0.88 | 4.46±0.94 |  | 2.21±0.62 | 2.61±0.84 |  | - | - |  | - | - |
| Garcés C [R19] | 482 | 784 |  | 0.82 ±0.29 | 0.82±0.3 |  | 4.73 ±0.75 | 4.74±0.73 |  | 2.79 ±0.64 | 2.83±0.72 |  | 1.55±0.36 | 1.53±0.33 |  | 1.37±0.2 | 1.36±0.19 |
| Birjmohun RS [R20] | 1293 | 1821 |  | - | - |  | - | - |  | 4.1±1.0 | 4.1±1.0 |  | 1.34±0.39 | 1.32±0.39 |  | 1.6±0.3 | 1.59±0.29 |
| Aydin M1 [R21] | 92 | 129 |  | 1.87 ± 1.01 | 1.56± 0.74 |  | 4.67± 1.07 | 4.64± 1.13 |  | 2.94± 0.88 | 2.98± 0.99 |  | 1.04±0.29 | 1.04±0.24 |  | - | - |
| Aydin M2 [R21] | 42 | 94 |  | 1.25 ± 0.51 | 1.15± 0.53 |  | 6.14± 1.37 | 5.13 ± 0.52 |  | 3.71± 0.79 | 3.24 ± 0.5 |  | 1.77±0.57 | 1.28±0.26 |  | - | - |
| Regieli JJ [R22] | 325 | 466 |  | - | - |  | 6.00± 0.86 | 6.09± 0.88 |  | 4.24± 0.76 | 4.36 ±0.81 |  | 0.94± 0.21 | 0.92 ±0.23 |  | - | - |
| Boesch-Saadatmandi C [R23] | 42 | 55 |  | - | - |  | - | - |  | 3.20 ±0.91 | 3.2± 0.85 |  | 1.48 ±0.32 | 1.55± 0.34 |  | 1.35±0.22 | 1.41±0.19 |
| Lakshmy R1 [R24] | 80 | 44 |  | - | - |  | - | - |  | - | - |  | 1.02± 0.26 | 1.04± 0.27 |  | - | - |
| Lakshmy R2 [R24] | 88 | 66 |  | - | - |  | - | - |  | - | - |  | 0.98± 0.26 | 0.96 ± 0.22 |  | - | - |
| Likidlilid A1 [R25] | 97 | 6 |  | 1.93±1.08 | 2.34±1.07 |  | 7.19±1.07 | 7.2±1.47 |  | 5.04±1.15 | 4.86±1.77 |  | 1.26±0.33 | 1.29±0.41 |  | - | - |
| Likidlilid A2 [R25] | 101 | 2 |  | 1.24±0.53 | 1.34±0.37 |  | 4.62±0.49 | 4.46±1.01 |  | 2.8±0.61 | 2.72±0.55 |  | 1.3±0.38 | 0.98±0.07 |  | - | - |
| Lenarcik A1 [R26] | 54 | 76 |  | 1.26+0.88 | 1.17+0.72 |  | 5.18+1.07 | 4.96+0.85 |  | 2.96+0.89 | 2.85+0.83 |  | 1.55+0.45 | 1.65+0.45 |  | - | - |
| Lenarcik A2 [R26] | 27 | 43 |  | 0.99+0.57 | 0.93+0.76 |  | 4.53+0.81 | 4.53+0.91 |  | 2.42+0.74 | 2.43+0.79 |  | 1.68+0.47 | 1.63+0.38 |  | - | - |
| Zafiropoulos A [R27] | 155 | 270 |  | 1.35 ± 0.82 | 1.51± 0.81 |  | - | - |  | - | - |  | - | - |  | - | - |
| Altuner D1 [R28] | 43 | 57 |  | 1.99 ± 0.85 | 1.74± 0.87 |  | 5.36 ± 1.27 | 5.21 ± 1.16 |  | 3.4 ± 1.07 | 3.23 ± 0.79 |  | 1.26± 0.23 | 1.25 ± 0.26 |  | - | - |
| Altuner D2 [R28] | 21 | 29 |  | 1.38± 0.66 | 1.35± 0.86 |  | 5.16 ±0.98 | 4.9± 1.08 |  | 3.19± 1.11 | 2.99 ±0.91 |  | 1.18 ±0.17 | 1.21± 0.24 |  | - | - |
| Cymbron T [R29] | 46 | 223 |  | 1.37±0.69 | 1.37 ±0.72 |  | 5.84±1.23 | 5.27±1.28 |  | 3.49± 0.88 | 3.26± 0.94 |  | 1.47±0.35 | 1.43± 0.35 |  | - | - |
| Haj Mouhamed D1 [R30] | 65 | 97 |  | - | - |  | - | - |  | - | - |  | 0.95± 0.23 | 0.94 ± 0.26 |  | - | - |
| Haj Mouhamed D2 [R30] | 76 | 62 |  | - | - |  | - | - |  | - | - |  | 1.07± 0.27 | 1.06 ± 0.27 |  | - | - |
| Moura LM [R31] | 74 | 244 |  | - | - |  | 6.31±1.59 | 6.54±2.01 |  | 3.75±1.38 | 4.33±1.42 |  | 2.05±0.25 | 1.38±0.33 |  | - | - |
| Asefi M1 [R32] | 37 | 63 |  | 1.31± 0.83 | 1.45± 0.71 |  | 4.42±0.96 | 4.29±1.19 |  | 3 ± 0.77 | 2.84 ± 1.05 |  | 1.15± 0.43 | 1.1 ± 0.37 |  | 1.39±0.57 | 1.4±0.53 |
| Asefi M2 [R32] | 47 | 53 |  | 1.31±0.56 | 1.51 ±0.82 |  | 4.14 ± 0.88 | 4.22± 1 |  | 2.77± 0.72 | 2.72 ± 0.73 |  | 1.17± 0.35 | 1.17 ± 0.36 |  | 1.45±0.45 | 1.57±0.53 |
| Kucuk ST [R33] | 43 | 92 |  | 1.69±0.09 | 1.83±0.57 |  | 5.15±1.2 | 5.28±1.13 |  | 3.17±0.91 | 3.42±1 |  | 0.97±0.29 | 0.98±0.21 |  | - | - |
| Kucuk ST [R33] | 47 | 63 |  | 1.41±0.08 | 1.27±0.22 |  | 5.3±1.06 | 5.36±1.25 |  | 3.28±0.91 | 3.38±1.15 |  | 1.29±0.38 | 1.25±0.41 |  | - | - |
| Bahrehmand F1 [R34] | 39 | 70 |  | 2.04±1.8 | 2±1.48 |  | 4.91±1.98 | 5.15±1.03 |  | 3±0.65 | 3.15±0.83 |  | 1.08±0.76 | 1.09±0.39 |  | - | - |
| Bahrehmand F2 [R34] | 42 | 61 |  | 1.92±1.38 | 1.98±1.31 |  | 4.94±0.85 | 4.84±1.32 |  | 2.17±1.11 | 2.28±0.87 |  | 1.13±0.31 | 1.09±0.27 |  | - | - |
| Fekih O1 [R35] | 33 | 83 |  | 0.70 ± 0.31 | 0.72± 0.29 |  | 4.12 ± 0.71 | 3.87± 0.62 |  | 2.35± 0.67 | 2.24 ± 0.57 |  | 1.43± 0.28 | 1.43 ± 0.35 |  | 1.32±0.16 | 1.31±0.21 |
| Fekih O2 [R35] | 29 | 62 |  | 0.59 ± 0.24 | 0.93± 0.59 |  | 3.00± 0 .59 | 3.45 ± 0.67 |  | 1.55± 0.46 | 1.93 ± 0.46 |  | 1.27± 0.53 | 1.2± 0.39 |  | 1.35±0.51 | 1.47±0.29 |
| Macharia M [R36] | 25 | 819 |  | 1.5 ±0.7 | 1.5±0.93 |  | 5.9±1.2 | 5.6±1.2 |  | 3.8 ±1.1 | 3.63±1.0 |  | 1.4 ±0.4 | 1.3 ±0.37 |  | - | - |
| Shao ZY [R37] | 523 | 62 |  | 1.88 ± 0.83 | 2.09± 0.94 |  | 4.38 ± 1.19 | 4.45± 0.98 |  | 3.35± 0.93 | 3.47 ± 0.83 |  | 1.20± 0.39 | 1.26 ± 0.49 |  | - | - |
| Abessolo FA1 [R38] | 97 | 94 |  | - | - |  | - | - |  | 4.32± 0.39 | 4.3± 0.44 |  | 0.94± 0.59 | 0.81 ± 0.24 |  | 0.85±0.05 | 0.70±0.03 |
| Abessolo FA2 [R38] | 65 | 90 |  | - | - |  | - | - |  | 4.45± 0.40 | 4.43 ± 0.64 |  | 0.74± 0.40 | 0.57 ± 0.17 |  | 1.11±0.02 | 0.79±0.05 |
| Abessolo FA3 [R38] | 32 | 63 |  | - | - |  | - | - |  | 4.19± 0.45 | 4.16 ± 0.49 |  | 0.65± 0.28 | 0.45± 0.14 |  | 0.92±0.02 | 0.52±0.03 |
| Abessolo FA4 [R38] | 106 | 54 |  | - | - |  | - | - |  | 3.47± 0.62 | 3.4 ± 0.31 |  | 1.64± 0.41 | 1.28± 0.28 |  | 1.3± 0.07 | 1.16±0.08 |
| Bounafaa A [R39] | 77 | 129 |  | 1.86±0.61 | 1.93±1.02 |  | 4.73±0.09 | 4.63±1.36 |  | - | - |  | - | - |  | - | - |
| Sayın Kocakap DB [R40] | 55 | 59 |  | 1.91±1.01 | 1.96±1.1 |  | 5.1±1.1 | 4.9±1.01 |  | 3.09±0.88 | 2.87±0.81 |  | 1.21±0.29 | 1.14±0.25 |  | - | - |
| Fridman O1 [R41] | 48 | 78 |  | 1.66±1.25 | 2.41±2.21 |  | 5.06±2.29 | 5.24±1.41 |  | 2.50±1.18 | 2.68±1.32 |  | 1.47±0.42 | 1.54±0.35 |  | - | - |
| Fridman O2 [R41] | 88 | 115 |  | 1.85±1.59 | 1.47±0.97 |  | 5.13±1.03 | 5.03±1.29 |  | 2.64±0.94 | 2.74±1.07 |  | 1.69±0.56 | 1.64±0.43 |  | - | - |
